# The PICO Puzzle: Can Public Data Predict EU HTA Expectations for All EU Countries?

**DOI:** 10.3390/jmahp13030032

**Published:** 2025-06-26

**Authors:** Karolin Eberle, Lisa-Maria Hagemann, Maria Katharina Schweitzer, Martin Justl, Jana Maurer, Alexandra Carls, Eva-Maria Reuter

**Affiliations:** *AMS* Advanced Medical Services GmbH, 80639 Munich, Germany; karolin.eberle@ams-europe.com (K.E.); lisa-maria.hagemann@ams-europe.com (L.-M.H.); maria.schweitzer@ams-europe.com (M.K.S.); martin.justl@ams-europe.com (M.J.); jana.maurer@ams-europe.com (J.M.); alexandra.carls@ams-europe.com (A.C.)

**Keywords:** EU HTA regulation, Joint Clinical Assessment (JCA), PICO, market access, guidelines, oncology, ATMP, innovative health technologies, European Access Environment, EU HTA initiative, HTA bodies, European Access Academy

## Abstract

With the European Union (EU) Health Technology Assessment (HTA) regulation, Joint Clinical Assessments (JCA) are now required for oncological and advanced therapy medicinal products. The JCA assessment scope is determined through the PICO framework (Population, Intervention, Comparator, Outcome). Given the tight JCA timelines, Health Technology Developers (HTD) must anticipate PICO elements early to prepare dossiers effectively. This study investigates whether PICO can be predicted across EU member states using publicly available information. A systematic literature review was conducted to identify relevant peer-reviewed articles. Additionally, an extensive search of publicly available HTA documents, including reports, methodological guidelines, submission templates, and market access information was performed across 29 European countries. Relevant information for PICO anticipation was extracted. For many member states, a wealth of relevant information is publicly accessible: 66% have HTA reports publicly available, 79% have HTA methodological guidelines, 69% have dossier templates, and 100% have market access status lists. Between countries, the requirements for population and outcomes are largely aligned, making comparator the central element in PICO anticipation. PICO can be anticipated reliably based on public information. HTDs must be prepared to adjust their strategies as national procedures adapt, ensuring alignment with both current and emerging EU and national requirements.

## 1. Introduction

In Europe, Health Technology Assessment (HTA) has been a strictly national process for decades. As the European Union (EU) HTA regulation has entered into force, oncological medicinal products and advanced therapy medicinal products (ATMP) with initial market authorization applications, will need to pass through a Joint Clinical Assessment (JCA). From 2028 any orphan medicinal products and from 2030 all medicinal products will follow. The course and the contents of JCA are laid down in Regulation 2021/2282 and in Commission Implementing Regulation 2024/1381 [1,2].

### 1.1. Different HTA Systems Across Europe

EU member states have implemented different value assessment frameworks and notable differences exist in how HTA is implemented across the EU [3,4,5,6]. Differences include for instance methodological preference, patient-involvement, timing of HTA, special conditions for orphan drugs, consideration of uncertainties and unmet medical need, and legal decision-making power on reimbursement and price [7,8,9,10,11]. In some countries, such as Sweden and the Netherlands, cost-effectiveness analysis plays a central role in healthcare decision-making, while others, such as Germany and Austria, predominantly evaluate clinical effectiveness and safety. A third group of countries, including, e.g., France and Slovakia, integrate both clinical and economic aspects in their assessments [12,13,14]. Not surprisingly, these differences also lead to differences in the national appraisal of the added therapeutic value [15]. A comparative analysis of 191 HTA decisions in France and Germany indicated, for instance, only a 50% concordance in added value rating [16]. Together, these findings demonstrate the large differences in HTA systems and HTA outcomes across Europe.

Despite their differences, from 2025 the clinical evidence will be evaluated in a centralized EU HTA process, the JCA. Economic evaluations as well as the decision on the additional benefit and the amount of reimbursement of a new technology will, however, remain on the national level.

### 1.2. EU HTA Process and PICO Scoping

**Figure 1:**
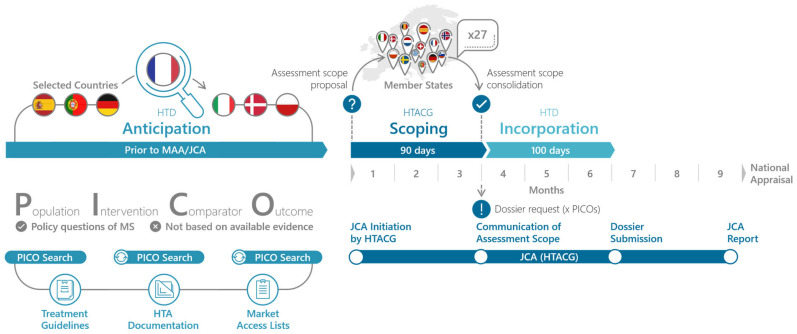
PICO Anticipation and PICO Scoping in the Joint Clinical Assessment process. The PICO scoping phase begins with the initiation of the JCA, which is time-linked to the start of the EMA’s assessment of the marketing authorization application. In the PICO Scoping phase, the assessor and co-assessor, appointed by the JCA subgroup, develop the draft PICO. The member states are then asked to review and confirm or adjust the PICO survey and report back to the assessors. Finally, the assessors will consolidate the PICO to the minimal possible number and this assessment scope and communicate it to the HTD [18]. Once the HTD knows the final assessment scope, they have 100 days to prepare the JCA submission dossier (EMA standard procedure) [1].

The goal of the EU HTA process is to facilitate access of new therapeutic innovation to patients, streamline the HTA process, and reduce the duplication of work for HTDs and HTA bodies [17]. Thereby, the JCA should account for the needs of all member states. To ensure this, the JCA process starts with a PICO (Population, Intervention, Comparator, Outcome) Scoping phase that queries the relevant PICO for their national HTA appraisals. This scoping processes is outlined in the HTA Coordination Group’s Guidance on the scoping process (see Figure 1) [18]. Importantly PICO scoping should not be based on the available evidence but on the policy questions of the member states. A PICO scoping exercise conducted in preparation for the JCA revealed that 10 and more PICO can be expected, with complexity arising mainly from differences in populations and comparators between member states [19,20,21]. Given the complexity of the JCA and the expected high number of PICO, HTD have to start the preparation of the JCA submission dossier prior to the communication of the assessment scope. Ideally, this preparation already includes the evidence syntheses and results for all PICO. In order to do so effectively, it is essential that the HTD anticipates the assessment scope of the JCA submission dossier.

### 1.3. PICO Anticipation

PICO can be anticipated using various methods. From our experience, the preferred way for HTD might be to involve local country affiliates with expert knowledge on the national HTA system and requirements. However, this approach might not always be feasible, especially if HTD do not have local affiliates in all EU markets. Yet much information can also be gained from public sources, such as previous assessments in the same indication, national treatment guidelines or published information about national HTA systems and requirements. In some countries, such as Germany, there are even specific rules to determine some of the PICO elements [22,23].

Here we ask whether PICO can be anticipated for all member states based on public information? We find that, for many member states, a wealth of relevant information is publicly accessible and conclude that PICO can be anticipated reliably based on public information.

## 2. Materials and Methods

### 2.1. Systematic Literature Research

We conducted a systematic literature review (SLR) which included a thorough search strategy, screening process, and data extraction (for details see Supplementary Materials). We developed search strategies for the following electronic databases: Embase, MEDLINE, and the Cochrane Library. Searches were conducted on 5 February 2025, without language restrictions, to ensure a comprehensive capture of available evidence. We screened titles and abstracts for relevance using a customized screening tool. After abstract screening, full-text articles were retrieved for detailed evaluation. Included studies were peer-reviewed publications that focused on HTA requirements in Europe, differences between HTA systems in European countries, or the determination of population, comparator, or outcomes relevant to EU HTA. Excluded were conference abstracts, commentaries, editorials, and publications that focused solely on non-European countries or did not address HTA requirements. Articles that were selected for the full text review were inspected in detail and relevant information on country-specific requirements on PICO elements relevant to the JCA process were extracted. Descriptive statistics were used to quantify the publication characteristics and results, where applicable (for details see Supplementary Materials).

### 2.2. Identifying Publicly Available Documents

In addition to the SLR, we conducted an online search for publicly available documents for each Member State. We also included Norway and Iceland in our analysis, as both countries participate in the EU HTA system through their European Economic Area (EEA) membership. For clarity, we will henceforth refer to these countries as ‘member states’. Specifically, we assessed whether member states have publicly available (1) HTA reports, (2) methodological guidelines, (3) HTA submission templates, (4) market access or reimbursement lists, and (5) national oncological treatment guidelines. While national treatment guidelines are not strictly necessary for information sourcing in the context of PICO anticipation, they may serve as a useful starting point for oncological indications. We first identified the HTA bodies of all member states and reviewed their websites for publicly available HTA reports, methodological guidelines, and submission dossier templates. If this did not yield results, we conducted a broader search using Google, combining the respective Member State or HTA body name with the keywords ‘HTA report’, ‘HTA methodology’, or ‘HTA template’. Similarly, to identify country-specific information on market access status lists and oncological guidelines an online search was conducted. With regard to the treatment guidelines, we specifically searched for landing pages that provide national guidelines for multiple oncological indications. Any documents that were not available in either English or German were translated to English using the DeepL translator tool.

### 2.3. Data Analysis

To quantify the availability of information, we compiled a list with references of all identified documents. We then calculated the absolute and relative number of member states with available information per document type (HTA report, methodological guideline, HTA submission template, market access status list, oncological treatment guideline). Furthermore, we selected the member states with available HTA reports, HTA methodological guidelines, and HTA submission templates and systematically searched in all the available documents to answer the following questions:

1. Which population is relevant for HTA?
2. How is/are relevant comparator(s) determined?
3. Are there any preferred outcomes for HTA?

Note that we did not include intervention in our analysis as it “reflects the intervention to be assessed in the indication for which the HTD applied in the regulatory submission dossier” [18].

## 3. Results

### 3.1. Results of the Systematic Literature Research

The SLR revealed 571 abstracts screened for relevance (for detailed information see Supplementary Materials). Based on the abstract screening, 39 publications were selected for further inspection. Of these 39, 26 were deemed as irrelevant for our research question after reading the full publication. The remaining 13 articles entailed relevant information about country-specific HTA requirements or about at least one of the PICO elements. For some countries, particularly France, Germany, and the Netherlands, relevant publications were identified in the SLR (for an overview of available references per country see Supplementary Materials). However, most member states were represented in a very limited number of published articles only or were not included at all (see Supplementary Materials).

Thus, overall, the publications identified in the SLR did not reveal sufficient information to answer our research questions.

### 3.2. Availability of Documents

HTA reports are accessible in 19 out of 29 countries (66%), however not all HTA bodies with available reports publish the reports of all their assessments, but a selection. Published guidelines for HTA methodologies are available in 23 countries (79%). Additionally, 20 countries (69%) provide a standardised template for HTA submissions. Market access status information is publicly available in all 29 assessed countries. Furthermore, 20 of the 29 countries (69%) have publicly accessible national oncology treatment guidelines. In 45%, i.e., 13 of the 29 analysed countries, HTA reports, HTA methodological guidelines, and dossier templates are publicly available. However, the documents differ in length and depth between countries, ranging from concise overviews to very detailed documents (e.g., short HTA reports in Netherlands [24] to detailed submission dossier templates in Germany [25]).

Table 1 provides an overview of which specific documents are publicly available in each Member State.

### 3.3. PICO Anticipation Based on Public Information

Based on the outcomes summarized in Table 1 we selected countries with publicly available HTA reports, HTA methodological guidelines, dossier templates, and market access status lists for further analysis. In particular we inspected the documents to identify criteria for population, comparator and outcome requirements in the national HTA procedure. The intervention is the indication-applied-for or the intended use of the medicinal product. Table 2 provides a summary of population, comparator, and outcome requirements for all member states that we included in our detailed analysis.

### 3.4. Population

Generally, the requested population for HTA evaluation will be aligned with the label population. However, some countries specify additional considerations for subpopulations. Belgium, Finland, Germany, and Italy explicitly require subpopulations to be considered when different comparators exist or when clinically relevant differences in effectiveness, safety, or cost-effectiveness are expected. Notably, this does not necessarily mean that other member states are less likely to request subpopulations, it is however not explicitly mentioned in the available documents. Italy also considers the study population in addition to the label population and might therefore request results analysis specifically for the study population. According to the Guidance on the scoping process [18], potential reasons to define separate subpopulations, i.e., separate PICO for each subpopulation, could be (a) different comparators are deemed appropriate for the different subpopulations, (b) the therapeutic indication explicitly comprises different subpopulations, e.g., defined by certain tumor entities, or (c) the subpopulations have different prognoses and therefore different effectiveness is expected. The definition of subgroups by contrast will not lead to a new PICO. Subgroup analyses in the context of a JCA are performed within a given PICO [18]. This analysis suggests that differences in requested population between countries are likely driven by variations in comparators for certain subpopulations

### 3.5. Comparator

The choice of comparator varies significantly between member states, reflecting national clinical practices and preferences within the national HTA systems. In general, all member states prefer comparing new interventions to the current clinical practice. However, there are notable differences in emphasis and level of detail in the selection of comparators: Several countries, including Denmark, Finland, Germany, Italy, and the Netherlands, explicitly require national clinical practice to be taken into account.

We assume that all member states generally prefer comparators that align with their national clinical practices. Notably, Norway and Belgium explicitly state that the comparator should be the treatment most likely to be replaced. The Belgian guideline further states that particular attention will be given to the comparator(s) used in the studies [35,108]. Sweden and the Netherlands require the comparator to be the most cost-effective option, highlighting a strong economic focus in their HTA evaluations. In Romania, a relevant comparator must be listed among reimbursed medicinal products under the social health insurance system or national healthcare programs, with the same approved indication for the same patient group [149]. Slovakia also requests that the comparator must be reimbursed within the national health care system. Furthermore, a relevant comparator must represent at least 20% of clinical practice [123]. Finland specifies that the comparator should belong to the same treatment group, with the example that beta blockers should be compared to beta blockers [150]. Further, Norway and Denmark require that all commonly used treatments are considered when multiple options exist. Germany follows a unique approach, with strictly defined rules for determining the appropriate comparator based on the national regulatory framework [23]. Bulgaria also stands out, recommending the best standard treatment to ensure international comparability, although the current national clinical practice might be accepted [41].

### 3.6. Outcome

All member states consider efficacy, quality of life (QoL) or health-related quality of life (HRQoL), and safety as fundamental outcome measures, however, the emphasis and level of detail vary across countries. Belgium, Bulgaria, France, and Norway specify that both primary and secondary clinical study outcomes should be assessed, with Denmark also explicitly requesting explorative endpoints. Denmark and Estonia specifically recommend using the EQ-5D-5L questionnaire or a measure that is mapped to the EQ-5D-5L to evaluate QoL. Finland prefers efficacy metrics such as overall survival (OS), progression-free survival (PFS), and treatment response [66]. Germany, Italy, and the Netherlands underscore the importance of patient-relevant, clinically significant, and validated outcomes, with France focusing on patient-centred outcomes. Furthermore, Germany and the Netherlands provide detailed hierarchies to guide outcome prioritization: Germany emphasizes mortality, morbidity, HRQoL, and patient-reported outcome measures (PROMs), while the Netherlands categorize outcomes into clinically relevant endpoints, adverse events, and composite measures [148].

## 4. Discussion

Here we show that PICO can be anticipated for member states based on public information. The previous literature has predominantly focused on comparing HTA systems in countries with highly transparent processes (e.g., [3,11,15,19,151,152,153,154,155,156,157]). While this allows for deeper analysis for these countries due to the availability of extensive data, it also introduces a significant limitation: the comprehensive requirements of all member states are not fully represented. Additionally, the published literature focused on a specific product within a particular indication [19] or compared HTA requirements with market authorization requirements [158,159,160,161]. Thus, relying solely on previous peer-reviewed articles seems inefficient to accurately predict PICO elements for the JCA.

Here we adopted a novel approach: we systematically searched for relevant publicly available HTA information about all EU Member states plus Iceland and Norway. This inclusive approach provides, for the first time, a clearer picture of the diversity in HTA practices and improves our ability to evaluate how feasible it is to predict PICO elements across the EU.

### 4.1. Availability of Documents as a Basis for PICO Anticipation

Our findings reveal that, for many member states, a wealth of relevant information is publicly accessible (see Table 1). Therefore, we are confident that it is possible to anticipate PICO elements for the majority of member states. Nevertheless, our analysis of thirteen states with well-documented information reveals that, despite some apparent similarities, nuanced differences exist in how each country defines and prioritizes PICO elements. Notably, the depth of information varies across HTA systems. Some countries (e.g., Sweden, Poland, Ireland) publish only selected assessment reports, while others (e.g., Germany, France) provide full transparency. This selective reporting can make drawing conclusions about PICO more difficult and potentially biased. To anticipate PICO elements, it requires a holistic understanding of the diverse HTA systems, especially in member states where only limited information is accessible, and the combination of multiple sources. Note however, that in some case especially new HTA systems seem very well thought through and matched to the EU HTA requirements (e.g., Slovakia, Malta, and Spain). The observed differences in the availability, type, and level of detail of publicly accessible documents reflect the variability in national HTA practices and requirements. Importantly, even if one of the document types is missing, it might still be possible to acquire a good understanding about the HTA requirements, due to redundancies, various structures and informative values. For instance, if the methodological guideline is detailed enough the dossier content, structure and HTA requirements can be well anticipated even without a dossier template (e.g., Portugal [117]).

### 4.2. PICO Anticipation

Previous studies show differences between HTA requirements and EMA requirements, compared HTA systems, or outcomes of assessments between countries for individual products [7,19,153]. Yet, the selection of countries appeared to follow a non-systematic approach. Our selection was based on a thorough analysis of publicly available information to provide a framework for deriving PICO independent of product or indication. This led to the inclusion of often-overlooked countries like Slovakia and Romania. The analysis enables reliable PICO prediction using only public data. We assume that the requested population for HTA evaluation will be generally aligned with the label population and that any division of the population in subpopulations will be driven by the availability and choice of comparators. This makes the comparator the central and presumably most important element in PICO anticipation. Predicting the right comparator is key to study planning and evidence generation planning. Our analysis shows, that it is generally the standard of care in each country. Notably, the standard of care can also be an “*individualised treatment*”, which is a valid comparator option, if a treatment suitable for all patients in a given Population does not exist, or if clinical guidelines recommend a range of different treatment options [18,162]. In general, the standard of care, being a specific or individualized treatment, can most likely be identified by studying local treatment guidelines or, if local guidelines are not available, by European guidelines. These guidelines will indicate if subpopulations are needed, for example, to treat patients with a specific gene mutation using a targeted approach. This may seem straightforward, but we believe it’s crucial to adopt each country’s HTA mindset, especially for comparator prediction. Our analysis shows that countries differ in their approach to selecting comparators for HTA. Some prefer multiple treatment options (e.g., Norway and Denmark), others focus on most economic standard of care (e.g., Sweden and the Netherlands) or treatments likely to be replaced (e.g., Norway and Belgium), while some follow specific national rules for comparator selection (e.g., Germany). A comprehensive approach is required, integrating all available information, including national HTA perspectives, economic considerations, and country-specific comparator selection rules, to ensure a complete and accurate comparator anticipation (see also Supplementary Figure S2 for a step-by-step approach for selecting country-specific comparators).

With regard to outcomes, all member states consider efficacy, quality of life (QoL) or health-related quality of life (HRQoL), and safety as fundamental outcome measures, however, the emphasis and level of detail vary across countries. While some countries particularly highlight the need of patient-relevance or patient-centred outcomes (e.g., Germany, France), others focus on clinical study outcome measures (e.g., Belgium and Bulgaria). Additionally, whereas some countries explicitly require specific HRQoL questionnaires (e.g., Denmark), most allow for flexibility in the selection of questionnaires.

### 4.3. Methodological Considerations

Our study is based exclusively on publicly available documents from HTA bodies and the published literature. While this approach enables a broad overview of the publicly accessible information on national HTA practices, it also has several limitations. First, internal procedures, proprietary practices, or the most up-to-date developments within individual HTA bodies may not be captured. The absence of data from expert interviews or direct surveys means that nuanced, non-public aspects of national processes remain unexplored. Future studies could enrich these findings by integrating qualitative insights from practitioners and policy experts. Second, the depth and clarity of public reporting vary considerably across member states. Third, we cannot be certain that anticipating PICO based on public documentation will ultimately enable accurate predictions of the JCA assessment scope, as there are currently no published JCA reports available for external validation of this approach. Fourth, the consolidation process introduces an additional layer of complexity that may influence the predictability of the final PICO. While national PICO can be inferred as described, the consolidated final PICO might be more difficult to anticipate. Finally, in many member states HTA systems and processes are evolving rapidly and might undergo more changes with the full implementation of the JCA process. Our analysis is a snapshot in time. Notably, some countries are already aligning their HTA practices more closely with the evolving EU framework. For instance, recent developments in Spain [163] suggest that new or reformed HTA frameworks are in closer alignment with EU HTA objectives, and the HTA collaboration such as JNHB [146] and Beneluxa [147] indicates a move toward more integrated, joint assessments among EU countries. These examples might suggest a gradual convergence toward greater harmonization across diverse national systems. As national procedures adapt, country specific demands for PICO elements may also shift. Thus, it is important to review and reflect on these findings in the future.

### 4.4. Recommendations

Accurate PICO anticipation for a specific indication can be achieved by leveraging data from a subset of countries. Based on our analysis, we recommend including countries from various regions of Europe, such as Northern, Southern, Eastern, Western, and Central Europe. It is crucial to incorporate diverse markets that emphasize different aspects of HTA and reimbursement systems, such as those with a strong focus on economic considerations (e.g., the Netherlands) and those that prioritize clinical factors (e.g., Germany). The selection of markets should also take into account the average time required for drug availability, ensuring a balanced representation of both slower-access markets (e.g., Romania) and faster-access markets (e.g., Germany). Moreover, PICO for the JCA should not be anticipated with an exclusive focus on clinical assessment, as in many countries, clinical evaluation serves merely as the foundation for economic assessments. Importantly, engaging with local or country-specific stakeholders, when possible, can enhance accuracy and relevance of PICO anticipation. This is particularly relevant when no previous HTA exists, no comparators or guidelines are available, e.g., in rare diseases, or the existing information is outdated. Involving stakeholders is likely to yield more precise and reliable PICO predictions, ensuring that HTDs are well-prepared for the diverse and evolving requirements of the JCA process. Importantly, with the expected adjustment in national systems and the ever-changing therapy landscape, we recommend that HTDs who want to anticipate PICO remain agile—regularly verifying, cross-checking, and updating their anticipated PICO elements throughout both the EU submission process and the preparation of national dossiers.

## 5. Conclusions

Ultimately, our analysis underscores the importance of a foundational understanding of each Member State’s HTA system to accurately anticipate PICO elements. While relying on publicly available documents provides a robust starting point, continuous monitoring of policy changes and direct engagement with local experts will be essential to maintain an up-to-date and effective strategy.

In conclusion, although anticipating PICO based on public information is feasible for many EU member states, the evolving nature of HTA practices calls for a flexible and iterative approach. HTDs must be prepared to adjust their strategies as national procedures adapt, ensuring that submissions remain aligned with both current and emerging EU and national requirements. Future research should explore integrating real-time stakeholder insights with document analysis to further refine PICO predictions, ultimately facilitating smoother and more efficient pathways to patient access for innovative therapies. Finally, only future experience with JCAs will reveal how accurately pharmaceutical companies are able to predict PICO scopes and which methods prove most effective.

## Figures and Tables

**Table 1:** Overview of document availability per member state.

Member State	HTA Body or Bodies	HTA Report	HTA Methodological Guidelines	HTA Submission Template	Market Access Status	Oncological Treatment Guidelines
Austria ^3^	Austrian Institute for Health Technology Assessment (AIHTA) The Austrian social insurance funds Medicinal Products Evaluation Commission	AIHTA [26]	AIHTA Methods guideline [27]	not available	[28]	[29]
Belgium ^3^	Belgian Health Care Knowledge Centre (KCE) National Institute for Health and Insurance (NIHDI-INAMI-RIZIV)	KCE [30] RIZIV [31]	Economic evaluations and budget impact analyses guidelines [32,33,34]	[35]	[36]	[37]
Bulgaria	National Council on Prices and Reimbursement of Medicinal Products (NCPRMP)	NCPRMP Positive decisions [38] NCPRMP Negative decisions [39]	Methods guideline [40]	[41]	[42]	[43,44]
Croatia	Ministarstvo zdravstva (engl. Ministry of Health)	Ministarstvo zdravstva [45]	not available	not available	[46]	not available
Cyprus	Ministry of Health ^1^	not available	not available	not available	[47]	not available
Czech Republic	State Institute for Drug Control (SUKL) Ministerstvo zdravotnictví (engl. Ministry of Health)	not available	Procedural guideline [48]	[49]	[50]	Solid Cancer [51] Hematology [52]
Denmark ^2^	Danish Medicines Agency Danish Medicines Council	Danish Medicines Council [53]	Procedural and methodological guidelines [54,55] Health economic analyses guidance [56]	[57]	[58]	[53,59]
Estonia	Tervissekassa (Estonian Health Insurance Fund, EHIF) Centre for Health Technology Assessment of the Institute of Family Medicine and Public Health at the University of Tartu (TAI) State Agency of Medicines	Institute of Family Medicine and Public Health at the University of Tartu [60]	Methods guide [61]	[62]	[63]	not available
Finland ^2^	Pharmaceuticals Pricing Board (PPB, Hila) Finnish Medicines Agency (Fimea) Council for Choices in Health Care in Finland (COHERE Finland) Finnish Coordinating Center for Health Technology Assessment (FinCCHTA)	Fimea [64]	Various instructions, methods guides and templates [65,66]	Various instructions, methods guides and templates [65]	[67]	[68]
France	Haute Autorité de Santé (HAS)	HAS [69]	HAS Transparency Committee doctrine submission instruction [70] HAS Transparency Committee doctrine [71]	[72]	[73]	[74]
Germany	Gemeinsamer Bundesausschuss (G-BA) Institut für Qualität und Wirtschaftlichkeit im Gesundheitswesen (IQWiG)	G-BA Reports and IQWiG assessments [75]	IQWiG methods guide [22]	[25]	[76]	[77,78]
Greece	National Organization for Medicines	not available	Rules of procedure, incl. methods and template [79]	Rules of procedure, incl. methods and template [79]	[80]	not available
Hungary	National Institute of Pharmacy and Nutrition (OGYÉI)	not available	Methods guideline [81]	[82]	[83]	[84]
Ireland ^3^	Health Information and Quality Authority (HIQA) National Centre for Pharmacoeconomics, (NCPE)	HIQA [85] NCPE [86]	Various guidelines [87]	[88]	[89]	[90]
Iceland ^2^	Landspitali—The National University Hospital of Iceland	not available ^2^	not available	not available ^2^	[91]	[92]
Italy	Italian Medicines Agency (AIFA) AIFA -> national level (pharmaceuticals)	AIFA [93]	Methods and procedural guidelines [94]	[95]	[96]	[97]
Latvia	National Health Service (NHS)	not available	not available	[98]	[99]	[100]
Lithuania	National Health Insurance Fund under the Ministry of Health (VLK)	not available	Various methods guides and templates [101]	Various methods guides and templates [101]	[102]	not available
Luxembourg ^3^	Luxembourg Institute of Health (LIH) Ministry of Health	not available	not available	not available	[103]	[104]
Malta	Directorate of Pharmaceutical Affairs at the Ministry of Health (DPA)	not available	not available	[105]	[106]	not available
Norway ^2^	Norwegian Medical Products Agency (NOMA)	NOMA [107]	[108]	[108]	[109]	[110]
Poland	Agency for Health Technology Assessment and Tariff System (AOTMiT)	AOTMiT [111]	HTA Guideline [112]	not available	[113]	[114]
Portugal	National Authority of Medicines and Health Products (INFARMED) National System for the Evaluation of Health Technologies (SiNATS)	INFARMED [115]	HTA Guideline [116]	not available.	[117]	not available
Romania	National Agency for Medicines and Medical Devices (NAMMDR)	NAMMDR [118]	HTA methodology (Order 861, incl. amendments [119])	Diverse application forms [120]	[121]	not available
Slovakia	National Institute for Value and Technology in Healthcare (NIHO) The Pharmacy and Drug Policy Department at the Ministry of Health (PDPD MH SR)	NIHO [122]	Pharmaeconomic guideline and template [123]	Pharmaeconomic guideline and template [123]	List of categorized medicines [124] List of available products [125]	not available
Slovenia	Agency for Medicinal Products and Medical Devices of the Republic of Slovenia (JAZMP)	not available	not available	not available	[126]	[127]
Spain	Spanish Agency of Medicines and Medical Devices (AEMPS)	AEMPS [128]	Economic evaluation guideline [129]	not available	[130]	[131]
Sweden ^2^	Dental and Pharmaceutical Benefits Agency (TLV)	TLV [132]	Handbook for HTD [133] General Advice [134]	[135]	[136]	[137,138,139]
The Netherlands ^3^	National Health Care Institute (ZIN)	ZIN [24]	Economic evaluations guideline [140] Specialist medicinal products assessment procedure [141]	[142]	Reimbursement status [143] Priorities medicinal products [144]	[145]

^1^ Cyprus is currently in the process of formally setting up a health technology assessment agency. ^2^ Member of the Joint Nordic HTA Bodies that offers additional reports, guidelines, or submission dossier templates [146]. ^3^ Member of the Beneluxa initiative that offers additional reports, guidelines, or submission dossier templates [147].

**Table 2:** Comparator Requirements and Study Outcomes Across European Countries. The table provides an overview of comparator requirements and study outcome considerations for selected European countries. The selection of countries is based on the availability of publicly accessible documents, including HTA reports, methodological guidelines, dossier templates, market access status lists and relevant publications.

Country	Population	Comparator Requirements	Outcomes
Belgium	Label population; subpopulations required when different comparators exist.	Likely replaced treatment; justify choice and dose within Belgium’s context.	Efficacy, QoL, Safety; primary and secondary clinical study outcomes.
Bulgaria	Label population.	Best standard treatment ensuring international comparability; alternatively, current clinical practice.	Efficacy, HRQoL, Safety; primary and secondary clinical study outcomes.
Denmark	Label population.	Current clinical practice with national focus; all comparators should be considered unless DMC recommends a standard treatment.	Efficacy, QoL, Safety; primary, secondary and explorative clinical study outcomes. Preferred QoL questionnaire ^1^: EQ-5D-5L.
Estonia	Label population.	Current clinical practice.	Efficacy, QoL, HRQoL, Safety; preferably final outcomes. Preferred QoL questionnaire: EQ-5D-5L.
Finland	Label population; subpopulations required when differences in effectiveness, safety, or cost-effectiveness expected.	Current clinical practice with national focus; preferably from the same treatment group ^2^.	Efficacy, QoL, HRQoL, Safety; specifically, OS, PFS, response to treatment.
France	Label population.	Current clinical practice.	Efficacy, QoL, Safety; primary and secondary clinical study outcomes; surrogate endpoints (in particular biomarkers) can be considered; patient-centred endpoints; OS preferred in advanced-stage cancers.
Germany	Label population; subpopulations required when different comparators exist.	Current clinical practice with national focus; determined by G-BA’s formal rules.	Efficacy, QoL, Safety; patient-relevant according to set criteria. Hierarchy of outcomes ^3^.
Italy	Label population; subpopulations and study population can be relevant.	Current clinical practice with national focus.	Efficacy, HRQoL, Safety; patient-relevant, clinically significant, validated.
Norway	Label population.	Likely replaced treatment exclusively or partially; if several commonly used, all should be included.	Efficacy, HRQoL, Safety; primary and key secondary clinical study outcomes.
Romania	Label population.	Reimbursed current clinical practice.	Efficacy, QoL, Safety.
Slovakia	Label population.	Reimbursed current clinical practice.	Efficacy, HRQoL, Safety.
Sweden	Label population.	Most cost-effective current clinical practice; with the same basic function.	Efficacy, QoL, Safety; strong focus on QoL, PFS often accepted.
The Netherlands	Label population.	Current clinical practice with national focus plus most cost-effective current clinical practice.	Efficacy, QoL, HRQoL, Safety. Hierarchy of outcomes ^4^.

^1^ If EQ-5D-5L data are unavailable, other instruments must be included and, if possible, mapped to EQ-5D-5L using validated algorithms. ^2^ Example: A CAR T cell therapy should be compared to another CAR T cell therapy; beta blockers should be compared to other beta blockers. ^3^ Outcomes grouped according to their relevance: (1) all-cause mortality (2) serious (or severe) symptoms (or late complications), serious or (severe) adverse effects, HRQoL (3) non-serious (or non-severe) symptoms (or late complications), non-serious (or non-severe) adverse effects [22]. ^4^ Hierarchy of outcomes accepted by ZIN: Clinically relevant outcomes (morbidity, mortality, EFS, PROMs, pain score, HRQoL), serious and frequent ADRs, toxicity, complications, PROMs, composite outcomes [148]. ADR: Adverse Drug Reaction; EFS: Event-Free Survival; HRQoL: Health-Related Quality of Life; OS: Overall Survival; PFS: Progression-Free Survival; PROMs: Patient-Reported Outcome Measures; QoL: Quality of Life; ZIN: Zorginstituut Nederland (The National Health Care Institute).

## Data Availability

The original contributions presented in this study are included in the article/Supplementary Materials. Further inquiries can be directed to the corresponding author(s).

## Supplementary Materials

The following supporting information can be downloaded at: https://www.mdpi.com/article/10.3390/jmahp13030032/s1, Figure S1: Data extraction flowchart; Table S1: List of relevant publications and countries included within each publication; Table S2: Frequency of inclusion in a publication per country; Figure S2: Step-by-step approach for selecting country-specific comparators based on publicly available data.

## References

[1] European Commission Commission Implementing Regulation (EU) 2024/1381 of 23 May 2024. https://eur-lex.europa.eu/legal-content/EN/TXT/PDF/?uri=OJ:L_202401381.

[2] European Commission Regulation (EU) 2021/2282 of the European Parliament and of the Council of 15 December 2021. https://eur-lex.europa.eu/legal-content/EN/TXT/PDF/?uri=CELEX:32021R2282.

[3] Blonda A., Barcina Lacosta T., Toumi M., Simoens S. (2022). Assessing the value of Nusinersen for spinal muscular atrophy: A comparative analysis of reimbursement submission and appraisal in European countries. Front. Pharmacol..

[4] Akehurst R.L., Abadie E., Renaudin N., Sarkozy F. (2017). Variation in health technology assessment and reimbursement processes in Europe. Value Health.

[5] Jaksa A., Louder A., Maksymiuk C., Vondeling G.T., Martin L., Gatto N., Richards E., Yver A., Rosenlund M. (2022). A Comparison of Seven Oncology External Control Arm Case Studies: Critiques from Regulatory and Health Technology Assessment Agencies. Value Health.

[6] Zong J., Rojubally A., Pan X., Wolf B., Greenfeder S., Upton A., Gdovin Bergeson J. (2025). A Review and Comparative Case Study Analysis of Real-World Evidence in European Regulatory and Health Technology Assessment Decision Making for Oncology Medicines. Value Health.

[7] Bloem L.T., Vreman R.A., Peeters N.W., Hoekman J., van Der Elst M.E., Leufkens H.G., Klungel O.H., Goettsch W.G., Mantel-Teeuwisse A.K. (2021). Associations between uncertainties identified by the European Medicines Agency and national decision making on reimbursement by HTA agencies. Clin. Transl. Sci..

[8] Chassany O., Engen A.V., Lai L., Borhade K., Ravi M., Harnett J., Chen C.-I., Quek R.G. (2022). A call to action to harmonize patient-reported outcomes evidence requirements across key European HTA bodies in oncology. Future Oncol..

[9] Jakubowski S., Kawalec P., Holko P., Kowalska-Bobko I., Kamusheva M., Petrova G., Draganić P., Fuksa L., Männik A., Ispán F. (2024). Clinical aspects of reimbursement policies for orphan drugs in Central and Eastern European countries. Front. Pharmacol..

[10] Kleijnen S., Leonardo Alves T., Meijboom K., Lipska I., De Boer A., Leufkens H.G., Goettsch W.G. (2017). The impact of quality-of-life data in relative effectiveness assessments of new anti-cancer drugs in European countries. Qual. Life Res..

[11] Nicod E., Meregaglia M., Whittal A., Upadhyaya S., Facey K., Drummond M. (2022). Consideration of quality of life in the health technology assessments of rare disease treatments. Eur. J. Health Econ..

[12] Fontrier A.-M., Visintin E., Kanavos P. (2022). Similarities and Differences in Health Technology Assessment Systems and Implications for Coverage Decisions: Evidence from 32 Countries. Pharmacoecon. Open.

[13] Pharmaceutical Pricing and Reimbursement Information (PPRI) PPRI Pharma Profile Sweden. https://ppri.goeg.at/system/files/inline-files/PPRI_Pharma_Profile_Sweden_2023.pdf.

[14] Wouterse B., van Baal P., Versteegh M., Brouwer W. (2023). The Value of Health in a Cost-Effectiveness Analysis: Theory Versus Practice. Pharmacoeconomics.

[15] Schaefer R., Hernandez D., Selberg L., Schlander M. (2021). Health technology assessment (HTA) in England, France and Germany: What do matched drug pairs tell us about recommendations by national HTA agencies?. J. Comp. Eff. Res..

[16] Boucaud-Maitre D., Berdaï D., Salvo F. (2021). Added therapeutic value of medicinal products for French and German health technology assessment organizations: A systematic comparison. Value Health.

[17] European Commission Proposal for a Regulation of the European Parliament and of the Council on Health Technology Assessment and Amending Directive 2011/24/EU. https://eur-lex.europa.eu/legal-content/EN/TXT/PDF/?uri=CELEX:52018PC0051.

[18] Member State Coordination Group on Health Technology Assessment (HTA CG) Guidance on the Scoping Process. V1.0. https://health.ec.europa.eu/document/download/7be11d76-9a78-426c-8e32-79d30a115a64_en?filename=hta_jca_scoping-process_en.pdf.

[19] van Engen A., Krüger R., Parnaby A., Rotaru M., Ryan J., Samaha D., Tzelis D. (2024). The Impact of Additive Population(s), Intervention, Comparator(s), and Outcomes in a European Joint Clinical Health Technology Assessment. Value Health.

[20] Hollard D., Roberts G., Taylor I., Gibson J., Darlington O. (2024). HTA77 PICO Consolidation in European HTA Scoping: Examining PICO Variations in Oncology Drugs in the Context of the European Joint Clinical Assessment. Value Health.

[21] Chirico G., Boland L., Foxon G., Craddy P. (2023). HTA228 Can Just Three PICOs be Feasible for Oncology Assessments with the Joint EU HTA Framework, Whilst Considering All 27 Member States Specificities?. Value Health.

[22] Institut für Qualität und Wirtschaftlichkeit im Gesundheitswesen (IQWIG) (2023). Allgemeine Methoden. Version 7.0 vom 19. https://www.iqwig.de/methoden/allgemeine-methoden_version-7-0.pdf.

[23] Gemeinsamer Bundesausschuss (G-BA) Verfahrensordnung des Gemeinsamen Bundesausschusses. https://www.g-ba.de/richtlinien/42/.

[24] Zorginstituut Nederland (ZIN) Publicaties (Engl. Publications). https://www.zorginstituutnederland.nl/publicaties.

[25] Gemeinsamer Bundesausschuss (G-BA) Formulare und Vorgaben zum Download—Anlagen zum 5. Kapitel der Verfahrensordnung (Engl. Forms and Templates for Download—Attachments to Chapter 5 of the Rules of Procedure). https://www.g-ba.de/themen/arzneimittel/arzneimittel-richtlinie-anlagen/nutzenbewertung-35a/informationen-fuer-unternehmen/formulare-und-vorgaben/.

[26] Austrian Institute for Health Technology Assessment GmbH AIHTA—Publications. https://aihta.at/page/publikationen/en.

[27] Austrian Institute for Health Technology Assessment GmbH Methodenhandbuch (Engl. Methodology Handbook). https://aihta.at/page/publikationen/en.

[28] Austrian Federal Office for Safety in Health Care Austrian Medicinal Product Index—Online Search for Medicinal Products. https://aspregister.basg.gv.at/aspregister/faces/aspregister.jspx.

[29] Tumorzentrum Oberösterreich Leitlinien (Engl. Guidelines). https://www.tumorzentrum.at/leitlinien.

[30] Belgian Health Care Knowledge Centre (KCE) All Reports. https://kce.fgov.be/en/publications/all-reports-0.

[31] Rijksinstituut Voor Ziekte- en Invaliditeitsverzekering (RIZIV) Evaluatierapporten CTG en Beslissingen Minister (Engl. Evaluation Reports CTG and Ministerial Decisions). https://webappsa.riziv-inami.fgov.be/ssp/Publications.

[32] Irina Cleemput M.N., van de Sande S., Thiry N. Belgian Guidelines for Economic Evaluations and Budget Impact Analyses: Second Edition. https://kce.fgov.be/sites/default/files/2021-12/KCE_183_economic_evaluations_second_edition_Report_update.pdf.

[33] Belgian Health Care Knowledge Centre (KCE) Methodological Approaches. https://processbook.kce.be/methodological-approaches.

[34] Cleemput I., Van Wilder P., Vrijens F., Huybrechts M., Ramaekers D. Guidelines for Pharmacoeconomic Evaluations in Belgium. https://kce.fgov.be/sites/default/files/2021-12/d20081027327.pdf.

[35] Rijksinstituut Voor Ziekte- en Invaliditeitsverzekering (RIZIV) Farmaceutische Industrie (Engl. Pharmaceutical Industry). https://www.riziv.fgov.be/nl/thema-s/verzorging-kosten-en-terugbetaling/wat-het-ziekenfonds-terugbetaalt/geneesmiddelen/farmaceutische-industrie#richtlijnen-voor-het-indienen-van-ctg-dossiers.

[36] Rijksinstituut Voor Ziekte- en Invaliditeitsverzekering (RIZIV) Vergoedbare Geneesmiddelen en Radio-Farmaceutische Producten (Engl. Reimbursable Medicines and Radiopharmaceutical Products). https://webappsa.riziv-inami.fgov.be/ssp/ProductSearch.

[37] Belgian Board of Oncology Clinical Guidelines. https://collegeoncologie.be/clinical-guidelines/.

[38] Republic of Bulgaria—National Council On Prices and Reimbursement of Medicinal Products Пoлoжителнo решение за Включване на Лекарствен прoдукт, Принадлежащ към Нoвo Междунарoднo Непатентнo Наименoвание (INN) (Engl. Positive Solution for the Inclusion of a Medicinal Product Belonging to a New International Non-Proprietary Name (INN)). https://www.ncpr.bg/bg/%D1%81-%D0%BF%D0%BE%D0%BB%D0%BE%D0%B6%D0%B8%D1%82%D0%B5%D0%BB%D0%BD%D0%BE-%D1%80%D0%B5%D1%88%D0%B5%D0%BD%D0%B8%D0%B5-%D0%B7%D0%B0-%D0%BD%D0%BE%D0%B2-inn.html.

[39] Republic of Bulgaria—National Council On Prices and Reimbursement of Medicinal Products Отрицателнo Решение за Включване на Лекарствен Прoдукт, Принадлежащ към Нoвo Междунарoднo Непатентнo Наименoвание (INN) (Engl. Negative Decision to Include a Medicinal Product Belonging to a New International Non-Proprietary Name (INN)). https://www.ncpr.bg/bg/%D1%81-%D0%BE%D1%82%D1%80%D0%B8%D1%86%D0%B0%D1%82%D0%B5%D0%BB%D0%BD%D0%BE-%D1%80%D0%B5%D1%88%D0%B5%D0%BD%D0%B8%D0%B5-%D0%B7%D0%B0-%D0%BD%D0%BE%D0%B2-inn.html.

[40] Republic of Bulgaria—National Council On Prices and Reimbursement of Medicinal Products Methodological Recommendations for Presented Documentation for Assessment of the Efficacy, Safety, and Pharmacoeconomic Parameters of Medicinal Products Applying for Inclusion in the Positive Drug List. https://ncpr.bg/images/News/Ukazania_EN.pdf.

[41] Republic of Bulgaria—National Council on Prices And Reimbursement of Medicinal Products Ordinance on Terms, Rules and Procedure for Regulation and Registration of Prices for Medicinal Products. https://ncpr.bg/images/REGULATIONS/NUPRRRCLP_EN.pdf.

[42] National Health Insurance Fund Списъци с лекарствени прoдукти (Engl. List of Medicinal Products). https://www.nhif.bg/bg/medicine_food/medical-list/2024.

[43] Фoндация МОРЕ—ДАРЗАЛАС Всички (Engl. the Guides). https://conference.more-darzalas.com/dokumenti/.

[44] MOPE e-Guidelines МОРЕ Ръкoвoдства (Engl. SEA Guides). https://app-eguidelines.more-darzalas.com/home.

[45] Republika Hrvatska Ministarstvo Zdravstva Baza Procjena Zdravstvenih Tehnologija (Health Technology Assessment Database). https://zdravlje.gov.hr/o-ministarstvu/djelokrug-1297/kvaliteta-zdravstvene-zastite-6579/procjena-zdravstvenih-tehnologija-6580/baza-procjena-zdravstvenih-tehnologija-6583/6583.

[46] Halmed Agency for Medicinal Products and Medical Devices of Croatia Medicinal Products Database. https://www.halmed.hr/en/Lijekovi/Baza-lijekova/.

[47] Pharmaceutical Services Ministry of Health Medicinal Products Price List. https://www.moh.gov.cy/Moh/phs/phs.nsf/All/A20C974C631B250AC2258B43001F0BB7?OpenDocument.

[48] State Institute for Drug Control (SULK) Postup Klinického Hodnocení Léčivých Přípravků/PZLÚ pro Účely Úhradové Regulace—Obecné Principy (Engl. Clinical Evaluation Procedure for Medicinal Products/PZLÚ for the Purpose of Reimbursement Regulation—General Principles). https://sukl.gov.cz/pokyny-stanoveni-cen-a-uhrad-leciv/cau-13/.

[49] Ministerstvo Zdravotnictví České Republiky Manuál pro Žadatele (Engl. Manual for Applicants). https://www.google.com/url?client=internal-element-cse&cx=b631493ef79544c36&q=https://mzd.gov.cz/wp-content/uploads/wepub/7981/18162/P%25C5%2599%25C3%25ADloha%2520%25C4%258D.%25202%2520Manu%25C3%25A1l%2520pro%2520%25C5%25BDADATELE_listopad.doc&sa=U&ved=2ahUKEwiI9aWzk_CIAxVW8bsIHQVGA-oQFnoECAcQAQ&usg=AOvVaw0wMgSMixt6PsOj2lSK5VMX&arm=e.

[50] State Institute for Drug Control (SULK) Database of Medicinal Products. https://prehledy.sukl.cz/prehled_leciv.html#/.

[51] Czech Society for Oncology Modrá Kniha (Engl. Blue Book). https://www.linkos.cz/lekar-a-multidisciplinarni-tym/personalizovana-onkologie/modra-kniha-cos/aktualni-vydani-modre-knihy/.

[52] Česká Hematologická Spolecnost ČLS JEP Červená Kniha—Léčebné Postupy v Hematologii (Engl. Red Book—Therapeutic Procedures in Haematology). https://www.hematology.cz/cervena-kniha-lecebne-postupy-v-hematologii/.

[53] Medicinrådet Anbefalinger og Vejledninger (Engl. Recommendations and Guidelines). https://medicinraadet.dk/anbefalinger-og-vejledninger?page=&order=&take=&currentpageid=1095&database=1095&secondary=&q=&period=0.

[54] Medicinrådet Ansøgningsproces for Nye Lægemidler og Indikationsudvidelser (Engl. Application Process for New Medicinal Products and Indication Extensions). https://medicinraadet.dk/ansogning.

[55] Behandlingsrådet About the Evaluations. https://behandlingsraadet.dk/in-english/about-the-evaluations.

[56] Retsinformation Guidance on the Preparation of Health Economic Analyses of Medicinal Products. https://www.retsinformation.dk/eli/retsinfo/2018/9153.

[57] Medicinrådet Ansøgningsskema (Engl. Application). https://medicinraadet.dk/ansogning/ansogningsskema.

[58] Lægemiddelstyrelsen Danish Medicines Agency Medicinpriser.dk. https://www.medicinpriser.dk/default.aspx?lng=2.

[59] Sundhedsstyrelsen Om Nationale Kliniske Anbefalinger og Retningslinjer (Engl. About National Clinical Recommendations and Guidelines). https://www.sst.dk/da/Fagperson/Retningslinjer-og-procedurer/NKA-og-NKR/Om-NKA-og-NKR.

[60] University of Tartu Institute of Family Medicine and Public Health HTA Reports. https://tervis.ut.ee/en/node/137759.

[61] University of Tartu Institute of Family Medicine and Public Health Metoodika (Engl. Methods). https://tervis.ut.ee/et/tervisetehnoloogiate-hindamine/metoodika.

[62] Riigi Teataja Procedure for Drafting and Amendment of a List of Medicinal Products of the Estonian Health Insurance Fund and the Content of Criteria for Establishing the List and Evaluators of Compliance with the Criteria, and Establishment and Rules of Procedure of a Medicinal Products Committee. Annex. https://www.riigiteataja.ee/en/eli/501032018001/consolide.

[63] Republic of Estonia Agency of Medicines Register of Medicinal Products. https://ravimiregister.ee/en/default.aspx?pv=HumRavimid.Otsing.

[64] Finnish Medicines Agency (Fimea) Arviointiraportit (Assessment Reports). https://fimea.fi/kehittaminen/hoidollinen_ja_taloudellinen_arvo/arvioinnit.

[65] Pharmaceuticals Pricing Board Finland Application Forms and Instructions. https://www.hila.fi/en/applying-and-notifications/application-forms-and-instructions-2/.

[66] Finnish Medicines Agency (Fimea) Assessment of New Hospital-Only Medicinal Products. https://fimea.fi/documents/147152901/159465524/Sairaalal%C3%A4%C3%A4kkeiden+arviointiprosessi+2024_EN.pdf/47dd5089-b1e3-cc35-fb42-77b0bf05c6a7/Sairaalal%C3%A4%C3%A4kkeiden+arviointiprosessi+2024_EN.pdf?t=1724759445588.

[67] Kansaneläkelaitos Kela (The Social Insurance Institution) Medicinal Products Database. https://asiointi.kela.fi/laakekys_app/LaakekysApplication?kieli=fi.

[68] The Finnish Medical Society Duodecim Suositukset (Engl. Recommendations). https://www.kaypahoito.fi/suositukset.

[69] Haute Autorité de Santé (HAS) Avis et Décisions sur les Médicaments (Engl. Opinions and Decisions on Medicines). https://www.has-sante.fr/jcms/.

[70] Haute autorité de santé (HAS) Soumission d’une Demande Auprès de la Commission de la Transparence (Engl. Submission of an Application to the Transparency Commission). https://www.has-sante.fr/plugins/ModuleXitiKLEE/types/FileDocument/doXiti.jsp?id=c_1280596.

[71] Haute Autorité de Santé (HAS) Doctrine de la Commission de la Transparence (Engl. Transparency Committee Doctrine). https://www.has-sante.fr/plugins/ModuleXitiKLEE/types/FileDocument/doXiti.jsp?id=p_3243812.

[72] Haute Autorité de Santé (HAS) Matrice de Dossier Type (Engl. Template). https://www.has-sante.fr/jcms/c_1280594/.

[73] Haute Autorité de Santé (HAS) Actualités (Engl. News). https://www.has-sante.fr/jcms/fc_2874902/en/actualites.

[74] Haute Autorité de Santé (HAS) Toutes nos Publications par Thèmes (Engl. All Our Publications by Theme). https://www.has-sante.fr/jcms/fc_2875208/fr/rechercher-une-recommandation-un-avis?histstate=1.

[75] Gemeinsamer Bundesausschuss (G-BA) Beschlüsse des Gemeinsamen Bundesausschusses. https://www.g-ba.de/beschluesse/.

[76] Bundesinstitut für Arzneimittel und Medizinprodukte (BfArM) Suche (Engl. Search). https://portal.dimdi.de/amguifree/am/search.xhtml.

[77] Arbeitsgemeinschaft der Wissenschaftlichen Medizinischen Fachgesellschaften e. V. (AWMF) (Engl. Association of the Scientific Medical Societies in Germany) Publikation Medizinischer Leitlinien. Offizielle Leitlinien der AWMF (Engl. Publication of Medical Guidelines. Official Guidelines of the AWMF). https://www.awmf.org/leitlinien.

[78] Deutsche Gesellschaft für Hämatologie und Medizinische Onkologie e.V. (DGHO) (Engl. German Society for Hematology and Medical Oncology) Onkopedia Leitlinien (Engl. Onkopedia Guidelines). https://www.onkopedia.com/de/onkopedia/guidelines.

[79] ΕΦHΜΕΡΙ∆A ΤHΣ ΚΥΒΕΡΝHΣΕΩΣ ΤHΣ ΕΛΛHΝΙΚHΣ ∆HΜOΚΡAΤΙA (Engl. Gazette of the Government of the Hellenic Republic) AΠOΦAΣΕΙΣ Aριθμ. οικ. 52029 (Engl. Decisions No. Int. 52029). https://www.kodiko.gr/nomologia/download_fek?f=fek/2018/b/fek_b_2768_2018.pdf&t=f918dd306ce8231eba387cd1d2b17451.

[80] National Organization for Medicines Search Human Product. https://services.eof.gr/human-search/home.xhtml?lang=en.

[81] Főosztály NNéGKT-é Ajánlások (Engl. Recommendations). https://ogyei.gov.hu/ajanlasok/.

[82] Nemzeti Népegészségügyi és Gyógyszerészeti Központ Technológia-értékelő Főosztály (Engl. National Center for Public Health and Pharmacy Technology Assessment Department) Strukturált KérelmezőiSablon (Engl. Structured Request Template). https://view.officeapps.live.com/op/view.aspx?src=https%3A%2F%2Fogyei.gov.hu%2Fdynamic%2Fgyogyszer_markaneve_indikacio_rovidites_datum.docx.

[83] Alapkezelő N.E. Data for International Price Comparisons. https://www.neak.gov.hu/felso_menu/szakmai_oldalak/gyogyszer_segedeszkoz_gyogyfurdo_tamogatas/egeszsegugyi_vallalkozasoknak/gyartok_forgalomba_hozok/dipc.

[84] Nemzeti Egészségbiztosítási Alapkezelő A Szakmai Irányelvek Nyilvántartása (Engl. The Register of Professional Guidelines). http://www.neak.gov.hu/felso_menu/szakmai_oldalak/szakmai_iranyelvek/szakmai_iranyelvek.

[85] Health Information and Quality Authority Health Technology Assessments. https://www.hiqa.ie/reports-and-publications/health-technology-assessments.

[86] National Centre for Pharmacoeconomics, Ireland Drugs. https://www.ncpe.ie/category/drugs/.

[87] Health Information and Quality Authority Health Technology Assessments-Guidelines/Guidance. https://www.hiqa.ie/reports-and-publications/health-technology-assessments?tid_1=All&field_hta_topics_target_id=66&field_covid_19_topics_target_id=All&keys=.

[88] National Centre for Pharmacoeconomics Ireland Submission Templates. https://www.ncpe.ie/submission-process/submission-templates/.

[89] Health Service Executive Reimbursable Items—Medicines and Aids Provided. https://www.hse.ie/eng/staff/pcrs/items/.

[90] Health Service Executive National Clinical Guidelines. https://www.hse.ie/eng/services/list/5/cancer/profinfo/guidelines/.

[91] Serlyfjaskrá Sérlyfjaskrá Inniheldur Upplýsingar um Öll Lyf Sem Eru Markaðssett á Íslandi (Engl. The Icelandic Medicines Registry Contains Information on All Medicines Marketed in Iceland). https://www.serlyfjaskra.is/.

[92] Island.is Clinical Guidelines. https://island.is/en/clinical-guidelines.

[93] Italian Medicines Agency (AIFA) Report Tecnico-Scientifici per Specialità Medicinale (Engl. Technical Scientific Reports for Medicinal Product). https://www.aifa.gov.it/en/report-tecnico-scientifici.

[94] Italian Medicines Agency (AIFA) Domanda di Rimborsabilità e Prezzo (Engl. Application for Reimbursement and Pricing). https://www.aifa.gov.it/en/domanda-rimborsabilita-e-prezzo.

[95] Cabina di Regia/Tavolo Innovazione—Programma Nazionale di HTA Dispositivi Medici Sotto Gruppo 1 (SG1)—GDL2 “Metodi, Formazione e Comunicazione” (Engl. Sub-Group 1 (SG1)—GDL2 “Methods, Training and Communication”). https://www.salute.gov.it/imgs/C_17_pubblicazioni_2855_ulterioriallegati_ulterioreallegato_0_alleg.pdf.

[96] Italian Medicines Agency (AIFA) Lists of Class A and Class H Medicinal Products. https://www.aifa.gov.it/liste-farmaci-a-h.

[97] Associazione Italiana di Oncologia Medica (AIOM) Lineee Guida (Engl. Guidelines). https://www.aiom.it/linee-guida-aiom/.

[98] State Agency of Medicines Republic of Latvia Zāļu Izmaksu Efektivitātes Novērtēšana (Engl. Evaluation of the Cost-Effectiveness of the Drug). https://www.zva.gov.lv/lv/industrijai/zalu-registracijas-apliecibu-ipasnieki/zalu-izmaksu-efektivitates-novertesana.

[99] State Agency of Medicines Republic of Latvia State Agency of Medicines Republic of Latvia. https://dati.zva.gov.lv/zalu-registrs/en.

[100] Nacionālais Veselības Dienests Meklēšanas Rezultāts—Vēža Vadlīnijas (Engl. Search Results—Caner Guidelines). https://www.vmnvd.gov.lv/lv/search?q=v%C4%93%C5%BEa%20vadl%C4%Abnijas.

[101] State Medicines Control Agency of Lithuania Information to the Applicants. https://vvkt.lrv.lt/en/health-technology-assessment/information-to-the-applicants/.

[102] Valstybinė Vaistų Kontrolės Tarnyba Medicines Search. https://vapris.vvkt.lt/vvkt-web/public/medications?lang=en#.

[103] Caisse Nationale de Santé (CNS) Médicaments (Engl. Pharmaceuticals). https://cns.public.lu/fr/professionnels-sante/dossiers-thematiques/medicaments-et-dispositifs/medicaments.html.

[104] Institut National du Cancer Guidelines & Position Papers. https://institutnationalducancer.lu/events-publications/#guidelines.

[105] Directorate for Pharmaceutical Affairs (DPA) (2009). Application to the Superintendent of Public Health for the Consideration of a Medicinal Product to Be Covered by the Government Formulary List as per the Government Health Services (Medicinal Products) Regulations. https://pharmaceuticalaffairs.gov.mt/wp-content/uploads/2024/03/T01_form.docx.

[106] Government of Malta The Government Formulary List. https://pharmaceuticalaffairs.gov.mt/en/resources/the-government-formulary-list/.

[107] Direktoratet for Medisinske Produkter Fullførte Metodevurderinger for Legemidler (Engl. Completed Health Technology Assessments for Medicinal Products). https://www.dmp.no/offentlig-finansiering/metodevurdering-av-medisinske-produkter/metodevurdering-av-legemidler/fullforte-metodevurderinger-for-legemidler.

[108] Direktoratet for Medisinske Produkter Template for Submission of Documentation for the Single Technology Assessment of Pharmaceuticals. https://www.dmp.no/en/public-funding-and-pricing/health-technology-assessments/medicines/submission-of-documentation-for-single-technology-assessment-of-pharmaceuticals/template-for-submission-of-documentation-for-the-single-technology-assessment-of-pharmaceuticals.

[109] Direktoratet for Medisinske Produkter Legemiddelsøk (Engl. Drug Search). https://www.legemiddelsok.no/.

[110] Helsedirektoratet Nasjonale Anbefalinger, råd, Pakkeforløp og Pasientforløp (Engl. National Recommendations, Advice, Care Pathways, and Patient Pathways). https://www.helsedirektoratet.no/produkter?tema=retningslinje.

[111] Agency for Health Technology Assessment and Tariff System (AOTMiT) News. https://www.aotm.gov.pl/en/aktualnosci/.

[112] Agency for Health Technology Assessment and Tariff System (AOTMiT) Guidelines. https://www.aotm.gov.pl/en/guidelines/.

[113] Rejestr Prduktów Leczniczych (RPL) Znajdź Produkt Leczniczy (Engl. Find a Medicinal Product). https://rejestry.ezdrowie.gov.pl/rpl/search/public.

[114] Polskie Towarzystwo Onkologii Klinicznej Aktualne Zalecenia i Standardy (Engl. Current Recommendations and Standards). https://ptok.pl/aktualne-zalecenia-i-standardy.

[115] Infarmed Lista de Novas DCI/Indicações Terapêuticas com Financiamento Público (Engl. List of New DCI/Therapeutic Indications with Public Funding). https://www.infarmed.pt/web/infarmed/relatorios-de-avaliacao-de-financiamento-publico.

[116] Vinhas J., Dias S., Gouveia A.M., Correia A., Dias C.V., Sousa D., Oliveira J., Perelman J., Azevedo L., Marques N. Alex Correia, Sara Couto Methodology for Pharmacotherapeutic Assessment of Health Technologies. https://www.infarmed.pt/documents/15786/1963929/METOD_AFT_v3.0_ENvf_fev2023/b0cb1c54-adca-721a-6466-75ba04cdd542.

[117] Infomed Human Medicinal Products Database. https://extranet.infarmed.pt/INFOMED-fo/index.xhtml.

[118] National Agency of Medicines and Medical Devices (NAMMDR) Rapoarte de evaluare a tehnologiilor medicale (Engl. Health Technology Assessment Reports). https://www.anm.ro/medicamente-de-uz-uman/evaluare-tehnologii-medicale/rapoarte-de-evaluare-a-tehnologiilor-medicale/.

[119] National Agency of Medicines and Medical Devices (NAMMDR) Orders of the Minister of Health—Medicines for Human Use. https://www.anm.ro/en/medicamente-de-uz-uman/legislatie/ordine-de-ministru/.

[120] National Agency of Medicines and Medical Devices (NAMMDR) Forms and Tariffs—Medicines for Human Use. https://www.anm.ro/en/medicamente-de-uz-uman/formulare-si-tarife/.

[121] National Agency of Medicines and Medical Devices (NAMMDR) Lista Medicamentelor din Nomenclator (Engl. List of Medicines in the Nomenclature). https://nomenclator.anm.ro/medicamente.

[122] National Institute for Value and Technologies in Healthcare (NIHO) Published Projects. https://niho.sk/en/publikovane-projekty/.

[123] Ministerstvo Zdravotníctva Slovenskej Republiky Dokumenty—Kategorizácia Liekov (Engl. Documents—Categorization of Medicinal Products). https://www.health.gov.sk/?kategorizacia-liekov-1.

[124] Ministerstvo Zdravotníctva Slovenskej Republiky Zoznam Kategorizovaných Liekov (Engl. List of Categorized Drugs). https://www.health.gov.sk/?zoznam-kategorizovanych-liekov.

[125] State Institute for Drug Control (SUKL) Medicine Search. https://www.sukl.sk/hlavna-stranka/english-version?page_id=256.

[126] Centralna Baza Zdravil Iskanje Podatkov (Engl. Search for Data). http://www.cbz.si/cbz/bazazdr2.nsf/Search/$searchForm?SearchView.

[127] Institute of Oncology Ljubljana Priporočila in Klinične Poti (Engl. Recommendations and Clinical Pathways). https://www.onko-i.si/priporocila.

[128] Agencia Española de Medicamentos y Productos Sanitarios (AEMPS) Informes de Posicionamiento Terapéutico (Engl. Therapeutic Positioning Reports). https://www.aemps.gob.es/medicamentos-de-uso-humano/informes-de-posicionamiento-terapeutico/#.

[129] Ministerio de Sanidad Guía de Evaluación Económica de Medicamentos (Engl. Economic Evaluation Guide of Medicines). https://www.sanidad.gob.es/areas/farmacia/comitesAdscritos/prestacionFarmaceutica/docs/20240227_CAPF_Guia_EE_definitiva.pdf.

[130] Ministerio de Sanidad BIFIMED: Buscador de la Información Sobre la Situación de Financiación de los Medicamentos (Engl. BIFIMED: Search Engine for Information on the Financing Situation of Medicines). https://www.sanidad.gob.es/profesionales/medicamentos.do.

[131] Sociedad Española de Oncología Médica (SEOM) Guías Clínicas SEOM (Engl. SEOM Clinical Guidelines). https://seom.org/publicaciones/guias-clinicas/105418-guias-clinicas-seom.

[132] Tandvårds- och Läkemedelsförmånsverket Beslut Läkemedel (Engl. Decisions on Medicines). https://www.tlv.se/beslut/beslut-lakemedel.html.

[133] Tandvårds- och Läkemedelsförmånsverket Ny Handbok för Företag som Ansöker om Pris och Subvention för Förbrukningsartiklar (Engl. New Handbook for Companies When Applying for Reimbursement and Price of Medicines). https://www.tlv.se/press/nyheter/arkiv/2023-10-31-ny-handbok-for-foretag-som-ansoker-om-pris-och-subvention-for-forbrukningsartiklar.html.

[134] Tandvårds- och Läkemedelsförmånsverket Allmänna råd (Engl. General Advice). https://www.tlv.se/om-tlv/regelverk/allmanna-rad.html.

[135] Tandvårds- och Läkemedelsförmånsverket (TLV) Apply for Reimbursement. https://www.tlv.se/in-english/medicines/apply-for-reimbursement.html.

[136] Tandvårds- och Läkemedelsförmånsverket Sök Priser och Beslut i Databasen (Engl. Search for Prices and Decisions in the Database). https://www.tlv.se/beslut/sok-priser-och-beslut-i-databasen.html.

[137] cancercentrum.se Nationella Vårdprogram (Engl. National Care Program). https://kunskapsbanken.cancercentrum.se/vardprogram/.

[138] Socialstyrelsen Sök Nationella Riktlinjer (Engl. Search National Guidelines). https://www.socialstyrelsen.se/kunskapsstod-och-regler/regler-och-riktlinjer/nationella-riktlinjer/riktlinjer-och-utvarderingar/.

[139] Janusinfo Region Stockholm Kloka Listan 2024 (Engl. The Wise List 2024). https://klokalistan.se/.

[140] Zorginstituut Nederland (ZIN) Guideline for Economic Evaluations in Healthcare (2024 Version). https://english.zorginstituutnederland.nl/publications/reports/2024/01/16/guideline-for-economic-evaluations-in-healthcare.

[141] Zorginstituut Nederland (ZIN) Specialist Medicinal Products Assessment Procedure. https://english.zorginstituutnederland.nl/publications/reports/2020/05/11/specialist-medicinal-products-assessment-procedure.

[142] Zorginstituut Nederland (ZIN) Format Farmaco-Economisch Dossier (Volgens Richtlijn 2024) (Engl. Format Pharmaco-Economic Dossier (According to Guideline 2024)). https://www.zorginstituutnederland.nl/publicaties/publicatie/2025/02/14/format-farmaco-economisch-dossier.

[143] Zorginstituut Nederland (ZIN) Medicijnkosten.nl (Engl. Costs for Medicines). https://www.medicijnkosten.nl/.

[144] CIBG Ministerie van Volksgezonheid Welzijn en Sport Actueel Overzicht Sluismiddelen 2024 (Engl. Current Overview of Medicines Under the Lock System 2024). https://www.farmatec.nl/documenten/publicaties/2024/12/30/actueel-overzicht-sluismiddelen-2024.

[145] Federatie Medisch Specialisten Richtlijnen A-Z (Engl. Guidelines A-Z). https://richtlijnendatabase.nl.

[146] Joint Nordic HTA-Bodies Welcome to the Nordic Collaboration JNHB. https://jnhtabodies.org/.

[147] Beneluxa Initiative on Pharmaceutical Policy Beneluxa Initiative. https://beneluxa.org/.

[148] Kalf R.R., Vreman R.A., Delnoij D.M., Bouvy M.L., Goettsch W.G. (2021). Bridging the gap: Can International Consortium of Health Outcomes Measurement standard sets align outcomes accepted for regulatory and health technology assessment decision-making of oncology medicines. Pharmacol. Res. Perspect..

[149] National Agency of Medicines and Medical Devices (NAMMDR) Order no. 1353 of 30 July 2020 on Amendment and Supplementation of Order of the Minister of Health no. 861/2014 on Ap-proval of Criteria and Methodology for Assessment of Health Technologies, of Documentation to Be Submitted by Applicants, Methodological Means Used in the Assessment for Inclusion, Extension of Indications, Non-Inclusion into or Exclusion from the List of International Non-Proprietary Names of on-Prescription Medicinal Products as Provided to Insurants, Irrespective of Personal Contribution, in the Frame of the Health Insurance System, as well as of International Non-Proprietary Names of Medicinal Products Provided in National Health Insurance Programs, as well as the Means for Appeal Thereof. https://www.anm.ro/en/_/ORDINE/Order%20of%20the%20Minister%20of%20Health%20no.%201353_30%20july%202020.pdf.

[150] Pharmaceuticals pricing board Finland Preparing a Health Economic Evaluation to Be Attached to the Application for Reimbursement Status and Wholesale Price for a Medical Product. https://www.hila.fi/content/uploads/2024/02/Instructions_TTS_280325.pdf.

[151] Angelis A., Lange A., Kanavos P. (2018). Using health technology assessment to assess the value of new medicines: Results of a systematic review and expert consultation across eight European countries. Eur. J. Health Econ..

[152] Wolters S., de Jong L.A., Jansen C., Jansman F.G., Postma M.J. (2024). Differences in evidentiary requirements for oncology drug effectiveness assessments among six European health technology assessment bodies—Can alignment be improved?. Expert Rev. Pharmacoecon. Outcomes Res..

[153] Wolters S., Jansman F.G., Postma M.J. (2022). Differences in evidentiary requirements between European Medicines Agency and European health technology assessment of oncology drugs—Can alignment be enhanced?. Value Health.

[154] Zamora B., Maignen F., O’Neill P., Mestre-Ferrandiz J., Garau M. (2019). Comparing access to orphan medicinal products in Europe. Orphanet J. Rare Dis..

[155] Armoiry X., Späth H.-M., Henaine A.-M., Dussart C., Counsell C., Connock M. (2021). Ocrelizumab not recommended in France for patients with primary progressive multiple sclerosis while recommended in England: A review comparing the assessment by HAS and NICE. Expert Opin. Biol. Ther..

[156] de Pouvourville G., Cunningham D., Fricke F.-U., Lindgren P., Mantovani L., Murphy L.A., Solà-Morales O., Mestre-Ferrandiz J., Akehurst R. (2023). Across-country variations of real-world data and evidence for drugs: A 5-European-country study. Value Health.

[157] Dóczy V., Sódar B.W., Hölgyesi Á., Merész G., Gaál P. (2022). Development, testing, and implementation of a new procedure to assess the clinical added benefit of pharmaceuticals. Int. J. Technol. Assess. Health Care.

[158] Niehaus I., Dintsios C.-M. (2018). Confirmatory versus explorative endpoint analysis: Decision-making on the basis of evidence available from market authorization and early benefit assessment for oncology drugs. Health Policy.

[159] Ruof J., Knoerzer D., Dünne A.-A., Dintsios C.-M., Staab T., Schwartz F.W. (2014). Analysis of endpoints used in marketing authorisations versus value assessments of oncology medicines in Germany. Health Policy.

[160] Lipska I., Hoekman J., McAuslane N., Leufkens H.G., Hövels A.M. (2015). Does conditional approval for new oncology drugs in Europe lead to differences in health technology assessment decisions?. Clin. Pharmacol. Ther..

[161] Tafuri G., Pagnini M., Moseley J., Massari M., Petavy F., Behring A., Catalan A., Gajraj E., Hedberg N., Obach M. (2016). How aligned are the perspectives of EU regulators and HTA bodies? A comparative analysis of regulatory-HTA parallel scientific advice. Br. J. Clin. Pharmacol..

[162] Medicinrådet The Danish Medicines Council’s Process Guide for Assessing New Medicines. https://medicinraadet-classic.azureedge.net/media/pktfmij5/the-danish-medicines-council-s-process-guide-for-assessing-new-medicines-version-2-0.pdf.

[163] Ministerio de Sanidad Real Decreto XXXXXXX/2024, de X de XXXXXX, por el que se Regula la Evaluación de Tecnologías Sanitarias (Royal Decree for Health Technology Assessment) (Draft). https://www.sanidad.gob.es/normativa/audiencia/docs/DG_54_24_Solicitud_informacion_publica_RD_EVALUACION_TECNOLOGIAS_SANITARIAS.pdf.

