# Supplementary material for: The PICO Puzzle: Can Public Data Predict EU HTA Expectations for All EU Countries?
Source: J Mark Access Health Policy. 2025 Jun 26;13(3):32. doi: 10.3390/jmahp13030032 (PMC12286018; doi:10.3390/jmahp13030032)
Supplement: Supplementary file 1 [file jmahp-13-00032-s001.zip › jmahp-3637024-supplementary.pdf]

Manuscript title: The PICO Puzzle: Can Public Data Predict EU-HTA Expectations for all EU Countries?

Supplementary Material

Search Strategies

Embase

**Database**	Embase Classic+Embase [emczd]
**Interface**	Ovid
**Date of search**	05.02.2025
**Time Segment**	1947 to 2025 February 03
**Filter**	

#	Search term	Result
1	europ*.mp.	897707
2	exp biomedical technology assessment/ or exp clinical assessment/ or (hta or health technology assessment or clinical assessment or benefit assessment).mp.	336939
3	1 and 2	13798
4	(joint clinical assessment or jca).mp.	1001
5	3 or 4	14669
6	exp health care policy/ or exp reimbursement/ or exp decision making/ or (pico or scoping or scope or hta requirements or evidence requirements or policy making or health policy or decision making).mp.	1094850
7	5 and 6	2579
8	exp antineoplastic agent/ or exp oncology/ or exp neoplasm/ or exp cancer patient/ or exp malignant neoplasm/ or exp carcinoma/ or exp sarcoma/ or exp lymphoma/ or exp leukemia/	8511226
9	(oncolog* or cancer or carcinoma or sarcoma or lymphoma or leuk?emia or neoplas*).mp.	6501715
10	exp gene therapy/ or exp cell therapy/ or exp tissue engineering/ or (atmp or tissue engineer* or gene therap* or cell therap*).mp.	515279
11	8 or 9 or 10	9466082
12	7 and 11	854
13	limit 12 to yr="2019 - 2025"	515

Medline

Database	Ovid MEDLINE(R) and Epub Ahead of Print, In-Process, In-Data-Review & Other Non-Indexed Citations, Daily and Versions 1946 to February 04, 2025 [ppezv];
**Interface**	Ovid
**Date of search**	05.02.2025
**Time Segment**	1946 to February 04, 2025;
**Filter**	

#	Search term	Result
1	europ*.mp.	463718
2	exp Technology Assessment, Biomedical/ or (hta or health technology assessment or clinical assessment or benefit assessment).mp.	54542
3	1 and 2	2120
4	(joint clinical assessment or jca).mp.	646
5	3 or 4	2753
6	(pico or scoping or scope or hta requirements or evidence requirements or policy making or health policy or decision making).mp.	572367
7	5 and 6	572
8	exp Neoplasms/ or exp Carcinoma/ or exp Sarcoma/ or exp Antineoplastic Agents/ or exp Lymphoma/ or exp Leukemia/	4745648
9	(oncolog* or cancer or carcinoma or sarcoma or lymphoma or leuk?emia or neoplas*).mp.	4800051
10	exp "Cell- and Tissue-Based Therapy"/ or exp Mesenchymal Stem Cells/ or exp Tissue Engineering/ or exp Cell Transplantation/ or exp Genetic Therapy/ or (atmp or tissue engineer* or gene therap* or cell therap*).mp.	543273
11	8 or 9 or 10	6094880
12	7 and 11	90

Cochrane

**Database**	EBM Reviews - Health Technology Assessment 4th Quarter 2016 [clhta]; EBM Reviews - NHS Economic Evaluation Database 1st Quarter 2016 [cleed];
**Interface**	Ovid
**Date of search**	05.02.2025
**Time Segment**	4th Quarter 2016; 1st Quarter 2016;
**Filter**	

#	**Search term**	**Result**
1	europ*.mp.	1021
2	exp Technology Assessment, Biomedical/ or (hta or health technology assessment or clinical assessment or benefit assessment).mp.	16385
3	1 and 2	183
4	(joint clinical assessment or jca).mp.	0
5	3 or 4	183
6	(pico or scoping or scope or hta requirements or evidence requirements or policy making or health policy or decision making).mp.	2317
7	5 and 6	16
8	exp Neoplasms/ or exp Carcinoma/ or exp Sarcoma/ or exp Antineoplastic Agents/ or exp Lymphoma/ or exp Leukemia/	5961
9	(oncolog* or cancer or carcinoma or sarcoma or lymphoma or leuk?emia or neoplas*).mp.	6121
10	exp "Cell- and Tissue-Based Therapy"/ or exp Mesenchymal Stem Cells/ or exp Tissue Engineering/ or exp Cell Transplantation/ or exp Genetic Therapy/ or (atmp or tissue engineer* or gene therap* or cell therap*).mp.	238
11	8 or 9 or 10	7033
12	7 and 11	6

Inclusion and Exclusion Criteria

We predefined exclusion criteria. Publication was included if they:

focused on HTA processes or requirements in Europe or describe differences between HTA systems,

describe how population, comparator, or outcomes relevant to HTA are determined,

were published in peer-reviewed journals.

Publications were excluded if they:

focused exclusively on non-European countries,

did not involve HTA processes,

were conference abstracts, commentaries, or editorials.

Screening and Study Selection

We screened titles and abstracts for relevance using a customized screening tool. Following abstract screening, full-text articles were retrieved for detailed evaluation.

Data Extraction and Analysis

All articles that were selected for the full text review were inspected by two reviewers and relevant information on country-specific requirements on PICO elements relevant to the JCA process and copied to an excel file.

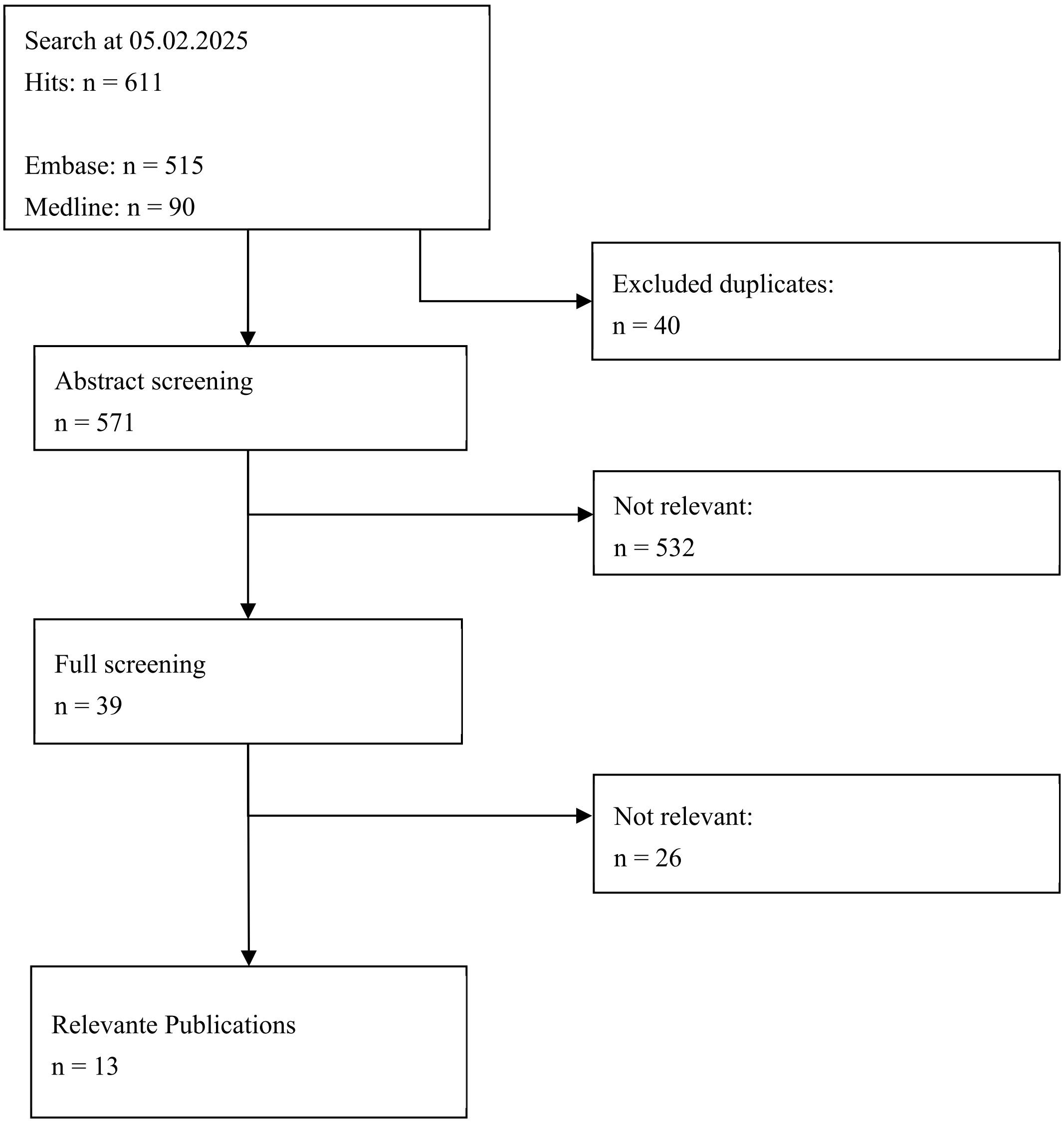

Supplementary Figure S1: Data extraction flowchart

Results

Supplementary Table S1: List of relevant publications and countries included within each publication

Publication	Countries included in the publication
[155]	France, England (Germany, Switzerland, Scotland)
[7]	France, Scotland, England/ Wales, Netherlands
[3]	Belgium, Canada, France, England and Wales, Germany, Italy, Ireland, Scotland, Sweden, the Netherlands, United States
[16]	France, Germany
[8]	Germany, France, UK
[156]	France, Germany, Italy, Sweden, England
[157]	Hungary
[9]	Bulgaria, Croatia, Czechia, Estonia, Hungary, Lithuania, Poland, Romania, Slovakia
[148]	Netherlands
[11]	Netherlands, France, Germany
[19]	Denmark, Germany, Netherlands, UK, France, Poland, Spain
[153]	Italy, The Netherlands, Poland, Portugal, England (together with Wales), Sweden
[152]	Italy, the Netherlands, Poland, Portugal, England and Wales, Sweden

Supplementary Table S2: Frequency of inclusion in a publication per country.

Country	Frequency
France	8
Netherlands	7
Germany	7
Sweden	4
Poland	4
Italy	4
Portugal	2
Hungary	2
Spain	1
Lithuania	1
Ireland	1
Estonia	1
Denmark	1
Czech Republic	1
Croatia	1
Bulgaria	1
Belgium	1

Flow chart on comparator section per country

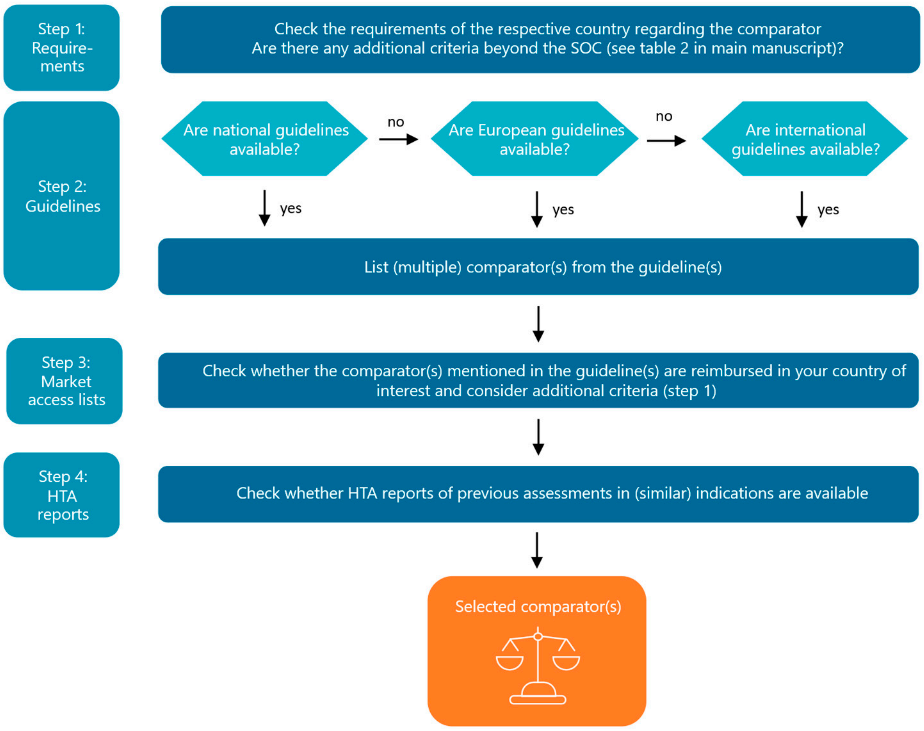

Supplementary Figure S2: Step-by-step approach for selecting country-specific comparators based on publicly available data. Note that if multiple comparators are identified across different clinical settings, this may affect the definition of relevant populations and, consequently, the PICO framework.
